# Genome-wide association mapping using mixed-models: application to GAW15 Problem 3

**DOI:** 10.1186/1753-6561-1-s1-s164

**Published:** 2007-12-18

**Authors:** Keyan Zhao, Magnus Nordborg, Paul Marjoram

**Affiliations:** 1 Molecular and Computational Biology Program, University of Southern California 1050 Childs Way Room 201B, Los Angeles, California 90089, USA; 2 Department of Preventive Medicine, University of Southern California, 1540 Alcazar Street, CHP 222, Los Angeles, California 90089, USA

## Abstract

We apply an analysis based upon mixed-models to the Genetic Analysis Workshop 15, Problem 3 simulated data. Such models are commonly used to mitigate the tendency for population structure, or cryptic relatedness, to inflate the false-positive rate of test statistics. They also allow for explicit modeling of varying degrees of relatedness in samples in which some individuals are related by (possibly unknown) pedigree, whereas others are not. Furthermore, the implementation of the method we describe here is quick enough to be used effectively on genome-wide data. We present an analysis of the data for Genetic Analysis Workshop 15, Problem 3, in which we show that these methods can effectively find signals in this data. Somewhat disappointingly, the false-positive rate does not appear to be reduced, but this is largely because the method used to simulate the data appears not to have encompassed effects, such as population stratification, that might have led to inflation of *p*-values.

## Background

A major issue when analyzing genome-wide data is that of false-positive signals. In part, this is caused by the large number of loci that are typically analyzed in such studies. It is often also caused by the effects of population stratification [1,2] or cryptic relatedness [3]. We focus on the second issue here. We apply mixed-model methods that have been developed to reduce the adverse effects of population structure, whether caused by geographical structure of populations, or relatedness (either observed or unobserved) between individuals.

Although the confounding effect of population structure can be minimized by careful matching design in case/control studies, there is still some evidence of confounding even in well designed studies [4]. Even when there is no evidence of stratification by standard methods, there can still be potential confounding. For example, in Campbell et al. [5] a SNP in the gene *LCT *that is totally unrelated to height showed strong association with height in a study in a European American population.

Two approaches that are commonly used to reduce the false-positive rate in the presence of population structure are genomic control [3] and structured association [6]. In other work we have shown that these do not deal with the problem in a satisfactory way [7,8], so we focus on a more recent approach: mixed models. This method has traditionally been applied in contexts such as cattle-breeding, in which extended pedigrees are known, or for a combined analysis of linkage and linkage-disequilibrium data, e.g., Meuwissen and colleagues [9-11]. More recently, Yu et al. [12] introduced a mixed-model approach suitable for application in a genome-wide context, in which relatedness was estimated via genome-wide marker data. In a recent paper, we extended this approach and applied it to data for *Arabidopsis thaliana *in which correlation between phenotypic distribution and population structure is high, and saw that the mixed-model approach greatly reduced the effects of population structure on false-positive rates [8].

The novelty in this paper is two-fold. First, we apply the method in a situation where the number of markers is much greater than in existing applications. Second, we apply the method in a context in which the degree of relatedness is known for some pairs of individuals (e.g., the affected sib-pairs) and unknown for others (e.g., pairs involving control individuals).

## Methods

We use a generalized linear mixed-model, which is an extension of Yu et al. [12]. We write

*η *= *Xα *+ *Zυ *+ *e*,

where *η *is the vector of linear predictors, which is related to the binary phenotype, affection status, through the inverse link function; *α *is a vector of fixed effects, corresponding to the SNP effects we are testing; *υ *is a vector of random effects reflecting the polygenic background; and *X*, *Z *are known incidence matrices relating the observations to fixed and random effects, respectively.

The mean and the variance of the model are assumed to be:

*E*[*η*] = *Xα*,

$$Var\left[\begin{array}{c} \upsilon \\ e \end{array}\right]=\left[\begin{array}{cc} K\sigma_{g}^{2} & 0\\ 0 & I\sigma_{e}^{2} \end{array}\right],$$

where K is the relationship matrix reflecting the genetic background correlations between individuals. (This is the term that reflects known or unknown relatedness information.) Using *y *to denote phenotype, the inverse link function for the probability of an individual being affected is the logit function:

$$P[y]=\frac{e^{\eta }}{1+e^{\eta }}.$$

We vary the way in which *K *is calculated in the model. First, because pedigree information is available, and controls are taken from different families and are assumed to be unrelated, we can use the known kinship to calculate *K *directly from the pedigree information. Second, in order to investigate how accurately kinship can be estimated when pedigree information is unknown, we can estimate the relative kinship [13] from genome-wide SNPs. For the purpose of comparison we also present results from a standard logistic linear regression analysis. We obtain this by removing the random effect term *Zυ *in Eq. (1).

## Results

A combined SNP data set with both of the 1500 ASP families and the 2000 unrelated control subjects is analyzed for each replicate. Because methods such as ours are widely used on SNP data, we analyze the SNP data only (9187 SNPs). Due to computational constraints, we only test the mixed model with estimated kinship (below) using a random subset of 50 ASP families and 200 controls. The Matvec package [14] was used in the analysis of combined data sets. The R package [15] and the glmmPQL library was used for generalized linear mixed models with estimated kinship. For the purposes of illustration, we focus on results for Replicate 1. We then present results across all 100 replicates in order to assess power.

**Figure 1:**
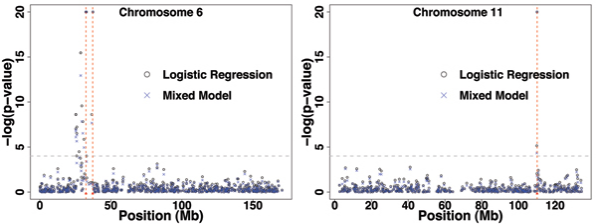
**Analysis of Replicate 1 from logistic regression and mixed-model analysis for chromosome 6 and chromosome 11**. The red lines denote the positions of the functional Loci C, D, and F, respectively. The horizontal dashed line corresponds to a *p*-value of 10^-4^.

In Figure 1 we show the results for both simple logistic regression and mixed-model analysis on chromosomes 6 and 11, respectively. In each case, the phenotype is RA (rheumatoid arthritis) affection status. We used the SNP genotypes only. The former does not explicitly allow for the relatedness between individuals, whereas the latter does. However, we see that for this data both analyses find strong signals in the correct locations. Indeed, the results of the two methods are extremely similar, due to the lack of population structure in the simulated data. Note that analysis of all regions other than chromosomes 6 (Loci C and D), 11 (Locus F), and 18 (Locus E) found no signal (results not shown.) The horizontal line corresponds to a *p*-value of 1 × 10^-4^, chosen because an examination of chromosomes with no functional loci rarely contained *p*-values below this number (see discussion of significance below). The results from models using estimated kinship give the same pattern.

**Figure 2:**
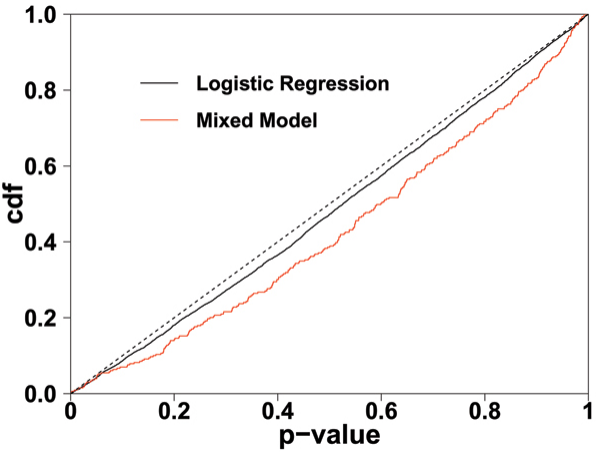
**Cumulative distribution function [cdf] of *p*-value distribution across all loci for Replicate 1**. The black line shows the distribution under a logistic regression analysis. The red line shows those resulting from the mixed-model analysis. The dashed line shows the ideal, theoretical null distribution.

These plots suggest that cryptic relatedness is not a problem for these data. To further examine this, in Figure 2, we look at the cumulative distribution function [cdf] of *p*-values across all null SNPs (i.e., SNPs on all chromosomes with no functional loci), for Replicate 1. This is similar to the idea in Papanicolaou et al. [16]. Compared to situations in which phenotype and genome-wide genotype are heavily correlated, where the cdf lies above the diagonal (e.g., Zhao et al. [8]), here the *p*-values are distributed close to the expected way. In fact they lie slightly below the diagonal, indicating under-dispersion. Rather than correcting the under-dispersion of the *p*-values, the mixed-model seems to exaggerate that tendency.

All replicates show the same pattern for the *p*-value distributions. Positive correlation between loci (i.e., linkage disequilibrium) can lead to under- or over-dispersion of *p*-values. We tested this possibility by thinning the data by choosing 1/10 of the SNPs. Now, the SNPs are no longer correlated (more than 95% of *r*^2 ^values between neighboring SNPs are less than 0.02). The dispersion pattern remains the same. Thus, correlation between loci is not the cause. The apparent tendency of a mixed-model analysis to exaggerate the under-dispersion has, to our knowledge, not been documented previously, and deserves further investigation to discover whether it is a consequence of the particular simulation scheme used for this data. Nonetheless, it is reassuring that signals are found on Loci C, D, E, and F despite this. Indeed, while mixed models are known to help when cryptic relatedness is present, our results indicate that they do not hurt a great deal when it is not.

To examine overall power, we analyzed all 100 replicates for the cases discussed above. We find that our method consistently finds the functional Loci C, D, E, and F in these regions. A formal power study would require a less heuristic method of assessing significance than an arbitrary (albeit supported by empirical results) definition of *p *< 10^-4^. One scheme is to proceed via a permutation-based test in which replicate data sets are created, their phenotypes permuted, and the distribution of smallest *p*-value is observed. Computational requirements prohibit the use of such a scheme here. In any case, the use of large numbers of genome-wide SNPs to get the null distribution and then derive an empirical *p*-value cut-off (10^-4 ^above), is probably sufficient. All 100 replicates have *p*-value less than 10^-4 ^at the SNP nearest to the functional Loci C, D, E, and F for the mixed-model analysis.

Finally, we assess the accuracy with which kinship is estimated using a pool of genome-wide markers. We find that results are good for the GAW15 simulated data. For kinship values within families that are supposed to be 0.25, estimates vary from 0.2 to 0.35. For values supposed to be 0, estimates vary from 0 to 0.05. Unfortunately, the simulated scheme appears not to have included any ancestry relationships between control individuals, or across case families. In reality, all individuals are related to some degree, although the significance of that relatedness will vary with individuals and with the analysis being performed. While our results show that kinship can be estimated in this study, a more comprehensive exploration of this issue, involving data with a more complex relatedness structure, would be of some interest.

## Discussion

These results show that a mixed-model scheme can be used successfully to analyze genome-wide SNP data. Unfortunately, the simulation scheme used for GAW15 has resulted in data with a range of relatedness that is not particularly rich. Thus, the data does not provide an ideal environment for assessing the performance of our method. This caveat is further strengthened by the fact that relatively simple methods, such as the logistic regression analysis we show here, also find the functional loci in the regions we have considered. Thus, while there is a growing body of evidence that mixed models provide an effective way of mitigating the negative effects of genome-wide correlation between genotypes (induced by factors such as population structure), the data here does not provide an ideal test for the method. In particular, the population of controls have been simulated in a way that results in little relatedness between them.

An obvious extension for future work is the detection of interactions. While there are a variety of problems associated with such analyses (see Marchini et al. [17]), the inclusion of interaction terms should be feasible within our framework. While the number of possible interactions is likely to be prohibitive for modern SNP chip data, a scheme in which only a small set are included in the model at any given moment, and in which we then explore the space of such models using a Markov-chain Monte Carlo scheme, should be feasible.

## Conclusion

We explored the feasibility of genome-wide association studies for binary traits using mixed models. In the simulated data we found no confounding due to population structure. Mixed models are flexible enough to be used in a context in which some individuals are related through known pedigree and others are not. While the results here provide an imperfect example of such an implementation, they provide cause for optimism in future studies.

## Abbreviations

ASP: affected sib pair

GAW: Genetic Analysis Workshop

SNP: single-nucleotide polymorphism

## Competing interests

The author(s) declare that they have no competing interests.

## References

[B1] Lander ES, Schork NJ (1994). Genetic dissection of complex traits. Science.

[B2] Marchini J, Cardon L, Phillips M, Donnelly P (2004). The effects of human population structure on large genetic association studies. Nat Genet.

[B3] Devlin B, Roeder K (1999). Genomic control for association studies. Biometrics.

[B4] Sladek R, Rocheleau G, Rung J, Dina C, Shen L, Serre D, Boutin P, Vincent D, Belisle A, Hadjadj S, Balkau B, Heude B, Charpentier G, Hudson TJ, Montpetit A, Pshezhetsky AV, Prentki M, Posner BI, Balding DJ, Meyre D, Polychronakos C, Froguel P (2007). A genome-wide association study identifies novel risk loci for type 2 diabetes. Nature.

[B5] Campbell CD, Ogburn EL, Lunetta KL, Lyon HN, Freedman ML, Groop LC, Altshuler D, Ardlie KG, Hirschhorn JN (2005). Demonstrating stratification in a European American population. Nat Genet.

[B6] Pritchard JK, Stephens M, Rosenberg NA, Donnelly P (2000). Association mapping in structured populations. Am J Hum Genet.

[B7] Aranzana MJ, Kim S, Zhao K, Bakker E, Horton M, Jakob K, Lister C, Molitor J, Shindo C, Tang C, Toomajian C, Traw B, Zheng H, Bergelson J, Dean C, Marjoram P, Nordborg M (2005). Genome-wide association mapping in *Arabidopsis *identifies previously known flowering time and pathogen resistance genes. PLoS Genet.

[B8] Zhao K, Aranzana MJ, Kim S, Lister C, Shindo C, Tang C, Toomajian C, Zheng H, Dean C, Marjoram P, Nordborg M (2007). An *Arabidopsis *example of association mapping in structured samples. PLoS Genet.

[B9] Meuwissen THE, Goddard ME (2001). Prediction of identity by descent probabilities from marker haplotypes. Genet Sel Evol.

[B10] Meuwissen THE, Karlsen A, Lien S, Olsaker I, Goddard ME (2002). Fine mapping of quantitative trait locus for twinning rate using combined linkage and linkage disequilibrium mapping. Genetics.

[B11] Meuwissen THE, Goddard ME (2004). Mapping multiple QTL using linkage disequilibrium and linkage analysis information and multitrait data. Genet Sel Evol.

[B12] Yu J, Pressoir G, Briggs WH, Vroh Bi I, Yamasaki M, Doebley JF, McMullen MD, Gaut BS, Nielsen DM, Holland JB, Kresovich S, Buckler ES (2006). A unified mixed-model method for association mapping that accounts for multiple levels of relatedness. Nat Genet.

[B13] Ritland K (1996). Estimators for pairwise relatedness and inbreeding coefficients. Genet Res.

[B14] Wang T, Fernando RL, Kachman SD Matvec User's Guide 2002. Version 1.03. http://statistics.unl.edu/faculty/steve/software/matvec/.

[B15] R Development Core Team R: A language and environment for statistical computing. http://www.R-project.org.

[B16] Papanicolaou G, Justice C, Kovac I, Sorant A, Wilson A (2005). Critical values and variation in type I error along chromosomes in the COGA dataset using the applied pseudo-trait method. BMC Genet.

[B17] Marchini J, Donnelly P, Cardon LR (2005). Genome-wide strategies for detecting multiple loci that influence complex diseases. Nat Genet.

